# MnO_2_-Modified Carboxylated Graphene Oxide Nanocomposite for the Effective Extraction of Organochlorine Pesticides from Environmental Water Samples

**DOI:** 10.3390/nano15231757

**Published:** 2025-11-23

**Authors:** Medhat A. Shaker, Wael H. Alshitari, Abeer H. Aljadaani, Faten M. Ali Zainy, Doaa S. Al-Raimi, Mustafa F. Mahmoud, Amel F. El Husseiny, Tarek E. Khalil, Amr A. Yakout

**Affiliations:** 1 Department of Chemistry, College of Science, University of Jeddah, Jeddah 21589, Saudi Arabia; 2 Chemistry Department, Faculty of Science, Damanhour University, Damanhour 22611, Egypt; 3 Cotton Spinning and Textile Industry Support Fund, Alexandria 21938, Egypt; 4 Chemistry Department, Faculty of Science, Alexandria University, Alexandria 21636, Egypt

**Keywords:** micro-solid phase extraction (µ-SPE), organochlorine pesticides, carboxylated-graphene oxide (CGO), manganese (IV) dioxide, gas chromatography–tandem mass spectrometry

## Abstract

A manganese dioxide-modified carboxylated graphene oxide (MnO_2_@CGO) nanocomposite was fabricated and utilized as a solid nanosorbent for extracting six organochlorine pesticides from environmental water samples. The target compounds, Hexachlorobenzene (HCB), β-Hexachlorocyclohexane (β-HCH), Heptachlor, Aldrin, Dieldrin, and o,p-Dichlorodiphenyltrichloroethane (o,p-DDT), were determined by micro-solid phase extraction (µ-SPE) coupled with gas chromatography–mass spectrometry (GC-MS) in selective ion monitoring mode. Key experimental factors influencing the extraction performance, such as sample pH, sorbent dosage, type and volume of eluting solvent, and time for desorption, were systematically optimized. Under the optimized conditions, the method showed good linearity (*R*^2^ = 0.998–1.000) within the concentration range of 0.1–5 ng L^−1^. The developed procedure was successfully applied to Nile River, agricultural wastewater, and groundwater samples, achieving recoveries between 87.1% and 101.2% with RSDs below 4.0%. The detection limits were 0.005–0.010 mg L^−1^ at a signal-to-noise ratio of 3.0. Overall, the MnO_2_@CGO-based µ-SPE method offers a sensitive, reliable, and straightforward approach for monitoring trace levels of organochlorine pesticides in environmental waters.

## 1. Introduction

According to the World Health Organization (WHO), around 800 million people lack access to a reliable source of drinking water, and approximately 830,000 deaths each year are linked to diarrheal diseases resulting from unsafe water, inadequate sanitation, and poor hygiene. This crisis is expected to worsen due to the combined effects of population growth, industrialization, climate change, and pollution, with nearly half of the global population projected to live in water-scarce regions by 2025 [1]. Consequently, efficient water treatment and disinfection methods are crucial to ensure the availability of safe water, particularly in low-income and rural communities.

Rapid industrial development has intensified global environmental challenges, especially water contamination by pharmaceuticals, pesticides, heavy metals, textile dyes, and surfactants [2]. Such pollutants pose severe health risks, including cancer, kidney and liver damage, and skin disorders [3]. Identifying and mitigating pollution sources are therefore essential to prevent further ecological harm [4]. Among the major persistent contaminants are organochlorine pesticides (OCPs), which have been widely used in agriculture since the 1940s for controlling insect-borne diseases such as malaria and typhoid [5,6]. Despite their effectiveness, OCPs are characterized by high toxicity, environmental persistence, and bioaccumulation, leading to long-term risks for aquatic ecosystems and human health [7,8]. Although many OCPs were banned in the 1970s, their use continues in some developing countries due to their low cost and high effectiveness [9,10,11,12,13,14,15,16,17,18]. Agricultural runoff, industrial discharge, and non-point pollution sources remain the principal routes of OCP entry into aquatic environments [19].

Due to their lipophilic and stable nature, the extraction of OCPs at ultra-trace levels is analytically challenging and requires efficient pre-concentration methods to overcome matrix interferences and enhance sensitivity [20,21,22,23]. Solid-phase extraction (SPE) has emerged as a superior alternative to traditional liquid–liquid extraction (LLE), offering reduced solvent consumption, shorter analysis time, lower cost, and improved reproducibility [24,25,26,27].

Recent advances in nanotechnology have introduced two-dimensional materials such as graphene (G), graphene oxide (GO), and carboxylated graphene oxide (CGO) as highly promising sorbents for water purification because of their large specific surface area, strong chemical stability, and negatively charged surfaces [28,29]. However, their performance can be hindered by sheet stacking and aggregation driven by π–π interactions and hydrophobic forces, which decrease accessible surface area and adsorption capacity [30,31]. To address these issues, graphene-based materials can be functionalized with metal or metal oxide nanoparticles to enhance dispersion, stability, and adsorption efficiency [32,33,34,35,36,37].

In this context, the present study reports the synthesis of an eco-friendly manganese dioxide-modified carboxylated graphene oxide (MnO_2_@CGO) nanocomposite and its application as an efficient sorbent for the extraction of six OCPs: hexachlorobenzene (HCB), β-hexachlorocyclohexane (β-HCH), heptachlor, aldrin, dieldrin, and o,p-dichlorodiphenyltrichloroethane (o,p-DDT), from environmental water samples. The MnO_2_@CGO nanocomposite was designed to combine the high adsorption capability of CGO with the surface complexation and catalytic properties of MnO_2_ nanoparticles. The π-electron-rich structure of CGO promotes strong π–π and hydrophobic interactions with OCPs, while MnO_2_ contributes to enhanced surface complexation and electron transfer processes, improving overall adsorption efficiency [38,39,40,41]. This work systematically optimizes experimental parameters, such as eluent type and volume, solution pH, sorbent dosage, and sample volume, to achieve maximum extraction performance. The reusability and stability of the MnO_2_@CGO nanosorbent are also examined. Comprehensive physicochemical characterization confirms the successful fabrication of the hybrid nanomaterial. Overall, this study presents a simple, cost-effective, and sustainable approach for the removal and determination of persistent OCPs from contaminated water, offering a potential solution for advanced environmental remediation and monitoring.

## 2. Materials and Methods

### 2.1. Chemicals and Reagents

**Figure 1:**
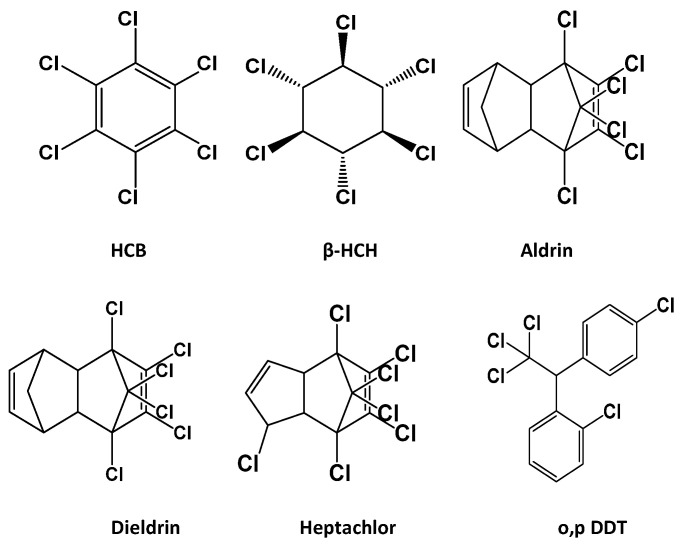
Chemical structures of the target OCPs.

Standards of Hexachlorobenzene (HCB), o,p′-Dichlorodiphenyltrichloroethane (o,p′-DDT), Aldrin, Dieldrin, β-Hexachlorocyclohexane (β-HCH), and Heptachlor (purity > 99.0%) (Figure 1) were supplied by Dr. Ehrenstorfer (Augsburg, Germany). High-purity solvents, including n-hexane, acetone, ethyl acetate, methanol (MeOH), and dichloromethane (HPLC grade), were purchased from SDFCL (SD Fine-Chem Limited, Mumbai, India), while acetonitrile was obtained from MACRON (Macron Fine Chemicals, Swedesboro, NJ, USA). Acetic acid (glacial, HPLC grade), sodium hydroxide, and phosphoric acid were supplied by Alpha Chemika (Mumbai, India). Hydrogen peroxide (HPLC grade), sodium nitrate, and ammonia solution (33%) were obtained from Sigma-Aldrich (Sigma Aldrich Chemicals Private Limited, Karnataka, India). Sulfuric acid (H_2_SO_4_) was purchased from EUROMEDEX (Souffelweyersheim, France), hydrochloric acid (HCl) from Research Lab (Research-Lab Fine Chem Industries, Mumbai, India), and sodium sulfite from LOBAChemie (Mumbai, India). All other chemicals used were of analytical grade. Standard stock solutions of each pesticide (1000 μg mL^−1^) were prepared in methanol. A mixed stock solution containing the six analytes at 100 μg mL^−1^ was prepared by diluting the individual stock solutions with MeOH. All prepared solutions were kept at 4 °C in the dark until further use.

### 2.2. Instrumentation

An Agilent 7890A gas chromatograph coupled with an Agilent 5975C mass selective detector (GC/MSD, Santa Clara, CA, USA) was used for the detection and quantification of organochlorine pesticides (OCPs). The separation was achieved on an HP-5MS capillary column (30 m × 0.32 mm i.d., 0.25 µm film thickness; Hewlett-Packard, Wokingham, UK). Helium (99.999% purity) served as the carrier gas. The Fourier Transform Infrared (FTIR) spectra of the samples were obtained using a spectrometer (ThermoFisher Scientific, Waltham, MA, USA) with the KBr pellet method over a wavenumber range of 400–4000 cm^−1^. The surface features were analyzed using a JEOL JEM-6010LV scanning electron microscope (Electron Optics Laboratory Co., Ltd., Tokyo, Japan) at magnifications between 5× and 300,000×, while detailed structural imaging was performed with a JEOL JEM-2100 high-resolution transmission electron microscope (Japan). Elemental composition was analyzed by energy-dispersive X-ray spectroscopy (EDX) using a QUANTA-250 instrument (ThermoFisher Scientific, Waltham, MA, USA). X-ray diffraction (XRD) patterns were recorded on a Shimadzu XRD-7000 (Tokyo, Japan) using Cu Kα radiation (λ = 1.5406 Å). UV-visible spectra of the nanosorbent suspensions were measured using a Thermo (USA) spectrophotometer (ThermoFisher Scientific, Waltham, MA, USA). Particle size and surface area (BET) analyses were conducted with a BEL Japan, Inc. analyzer (Osaka, Japan). Micro-solid phase extraction (µ-SPE) procedures were carried out using a 12-port SPE vacuum manifold (Supelco, Bellefonte, PA, USA).

### 2.3. Synthesis of CGO and MnO_2_@CGO

#### 2.3.1. Synthesis of CGO

Graphene oxide (GO) was synthesized from graphite powder using a modified Hummers method [42]. In a typical procedure, 1.0 g of graphite powder was dispersed in 60 mL of concentrated H_2_SO_4_ and stirred in an ice bath for 15 min. Then, 5.0 g of K_2_S_2_O_8_ and 3.0 g of P_2_O_5_ were added, and the mixture was magnetically stirred at 70–80 °C for 24 h. After cooling, 25.0 mL of H_2_SO_4_ and 3.0 g of KMnO_4_ were introduced to the pre-oxidized graphite, and the reaction was continued for 12 h. The mixture was then cooled in an ice bath, followed by the addition of 50 mL of water and 15 mL of H_2_O_2_, and kept for 30 min. The resulting product was filtered and washed with 0.5 mol L^−1^ HCl, yielding a black-brown solid of GO. To obtain CGO, 5.0 g of GO was sonicated in water until a uniform suspension was formed. Then, 30.0 g of NaOH and 25.0 g of chloroacetic acid were added, and the mixture was sonicated for 5 h to introduce carboxyl groups on the GO surface [43]. The resulting CGO was collected by filtration, thoroughly rinsed with water and methanol, and then vacuum-dried at 70 °C.

#### 2.3.2. Synthesis of MnO_2_@CGO Nanocomposite

Carboxylated graphene oxide (CGO) (5.0 g) was dispersed in 150 mL of absolute ethanol and sonicated until a uniform and stable suspension was obtained. Separately, 2.0 g of KMnO_4_ was dissolved in 100 mL of distilled water and transferred to a burette. The KMnO_4_ solution was then added slowly, dropwise, to the continuously stirred CGO suspension. A blackish-brown precipitate formed immediately, indicating the deposition of MnO_2_ onto the CGO surface. The resulting manganese dioxide-functionalized carboxylated graphene oxide (MnO_2_@CGO) nanocomposite was collected by filtration, thoroughly washed with water and ethanol, and dried under vacuum at 60 °C.

### 2.4. Sample Collection and Preparation

Tap water samples were freshly collected in the laboratory and stored in clean amber glass bottles. To remove residual chlorine, 5.0 mL of 0.50 mg L^−1^ sodium sulfite solution was added to each liter of collected tap water. River water samples were collected from three different locations along the Nile River using PVC containers, which were immediately sealed after gathering. All collected water samples were passed through 0.45 µm membrane filters using a Büchner funnel to eliminate suspended particles. Agricultural wastewater samples (12 L in total) were collected from two separate sites in agricultural areas located in Abees and Kafr El-Dawaar, Alexandria, Egypt. After collection, all samples were stored at 4 °C in the dark until further analysis.

### 2.5. Optimization of µ-SPE Conditions

Several experimental parameters influencing the performance of the µ-SPE procedure for OCPs were investigated and optimized. These included the sample pH, the amount of MnO_2_–CGO nanocomposite, the kind and volume of the elution solvent, as well as the volume of the sample. The extraction recovery (%R) of OCPs was calculated using the following equation:

$$\%\;R=\;\frac{C\;\cdot V}{C_{0\;\;}\cdot V_{0}}\;\times 100$$

where *C* represents the OCPs concentration (ng mL^−1^) in the reconstituted solvent, *C*_0_ is the initial concentration of OCPs in the water sample, *V* is the volume of the reconstituted solvent, and *V*_0_ is the volume of the water sample. For extraction, 50.0 mg of MnO_2_@CGO nanosorbent was manually packed into an 8.0 mL SPE glass cartridge fitted with frits. The cartridge was conditioned in a 12-port vacuum manifold by passing 3.0 mL of methanol twice, followed by 10.0 mL of deionized water. To study the effect of sample pH, five aqueous solutions (10 mL each) containing 1.20 µg HCB, 3.00 µg β-HCH, 1.25 µg Aldrin, 0.50 µg Dieldrin, 0.50 µg o,p-DDT, and 0.50 µg Heptachlor were prepared and adjusted to pH 3.00, 5.00, 6.80, 8.20, and 10.00 using 0.001 *M* H_2_SO_4_ or 0.001 *M* NaOH. Each solution was loaded onto the conditioned cartridge, and the retained analytes were eluted with 12 mL of dichloromethane (DCM). To determine the optimal sample volume, six aqueous samples (5–70 mL) containing 2.0 µg HCB, 3.0 µg β-HCH, 5.0 µg Dieldrin, 5.0 µg o,p-DDT, 12.0 µg Heptachlor, and 1.5 µg Aldrin were prepared at pH 5 and loaded through the cartridge. The retained OCPs were eluted with 12 mL of DCM. The choice of eluent solvent was evaluated using acetone, dichloromethane, ethyl acetate, n-hexane, and methanol. Ten milliliters of sample solution containing 75 ng HCB, 25 ng β-HCH, Heptachlor, and o,p-DDT, as well as 20 ng Aldrin and Dieldrin, were adjusted to pH 7 and extracted using 3 mL of each solvent. To optimize the eluent volume, the extraction was repeated using 10 mL aqueous solutions (pH 5) containing 1.25 µg HCB, 0.75 µg Aldrin, Dieldrin, and o,p-DDT, and 2.00 µg β-HCH and Heptachlor. Different volumes of DCM (3, 5, 7, 10, 12, 15, and 17 mL) were tested. Finally, method accuracy and precision were assessed at two concentration levels (2500 and 250 ng L^−1^) under the optimized µ-SPE conditions using MnO_2_–CGO nanosorbent at pH 5. Each concentration was extracted in five replicates and examined using GC-MS operated in selective ion monitoring (SIM) mode. All recoveries of the extracted OCPs were calculated based on GC-MS data.

## 3. Results and Discussion

### 3.1. Characterization of the MnO_2_@CGO Nanocomposite

**Figure 2:**
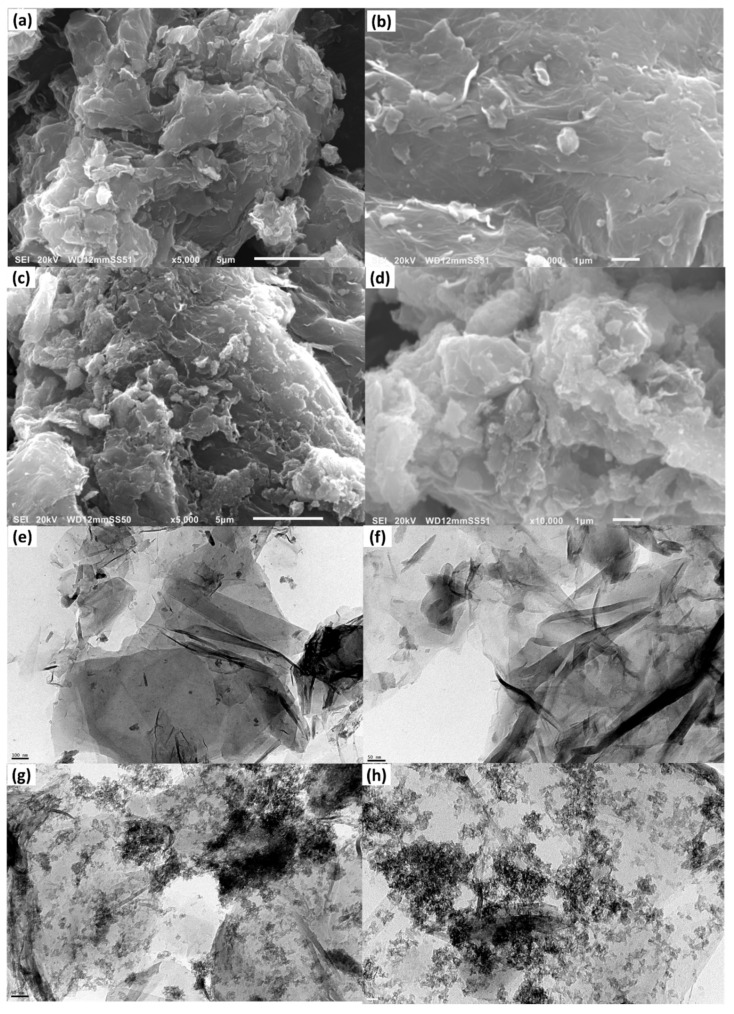
SEM (**a**,**b**) and (**c**,**d**) and HRTEM (**e**,**f**) and (**g**,**h**) of CGO and MnO_2_@CGO nanocomposites at different magnifications, respectively.

The surface morphology and internal nanostructure of CGO and MnO_2_@CGO nanocomposites were examined using SEM and HRTEM, as illustrated in Figure 2. The SEM micrographs of CGO (Figure 2a,b) display the typical wrinkled and layered structure characteristic of the carboxylated GO sheets. The surface appears relatively smooth and stacked, suggesting that partial restacking occurred during the drying process. This morphology confirms the preservation of the 2D lamellar nature of GO after carboxylation, with sheet-like layers loosely packed and interlinked through van der Waals forces. The folds and wrinkles observed on the CGO surface indicate good mechanical flexibility and the presence of numerous oxygenated functional groups (–COOH, –OH, and –CO), which increase the active surface area and enhance its hydrophilicity.

In contrast, the MnO_2_@CGO nanocomposite (Figure 2c,d) reveals a distinctly rougher and more compact texture. The originally smooth CGO layers became heavily decorated with uniformly distributed nanoparticles, confirming the successful anchoring of MnO_2_ on the CGO surface. The MnO_2_ nanoparticles appear as bright granular clusters covering the CGO sheets, which disrupt the stacking and create an irregular porous structure. This morphological transformation indicates strong interfacial interaction between MnO_2_ and the oxygenated functional sites of CGO, preventing sheet aggregation and improving dispersion stability. The high-magnification image (Figure 2d) further shows fine MnO_2_ nanocrystals embedded across the wrinkled CGO matrix, forming a heterogeneous hybrid surface favorable for adsorption processes. Such an arrangement not only increases the number of active binding sites but also enhances electron transport and surface complexation capabilities, essential for efficient extraction of OCPs.

The HRTEM images provide deeper insight into the internal architecture of the synthesized materials. As seen in Figure 2e,f, the CGO exhibits thin, transparent, and nearly smooth nanosheets with slight folding at the edges, reflecting a high degree of exfoliation and sheet integrity. These sheets are mostly free from particulate deposition, corroborating the purity and monolayer character of the CGO.

Upon modification with MnO_2_, the TEM micrographs (Figure 2g,h) clearly reveal dense distributions of dark, well-defined MnO_2_ nanoparticles anchored to the CGO layers. The nanoparticles are uniformly dispersed without significant agglomeration, implying efficient nucleation and growth on the functionalized carbon surface. The particle sizes of MnO_2_ are mostly below 20 nm, indicating nanoscale dispersion. The contrast difference between the dark MnO_2_ spots and the lighter CGO background confirms the successful hybridization of both components. The fine dispersion of MnO_2_ nanoparticles within the CGO matrix creates abundant mesopores and heterogeneous adsorption domains, which are expected to facilitate rapid mass transfer of target analytes during extraction.

Overall, the SEM and HRTEM analyses collectively demonstrate that MnO_2_ NPs were successfully incorporated onto the CGO surface, forming a 3D, rough, and porous nanocomposite structure. The synergistic combination of MnO_2_ and CGO provides an enlarged surface area, abundant oxygen-containing functional groups, and multiple adsorption sites. These features are anticipated to significantly enhance the affinity of MnO_2_@CGO toward organochlorine pesticides through π–π interactions, hydrogen bonding, and surface complexation, ultimately contributing to its superior extraction performance observed in subsequent analytical experiments.

**Figure 3:**
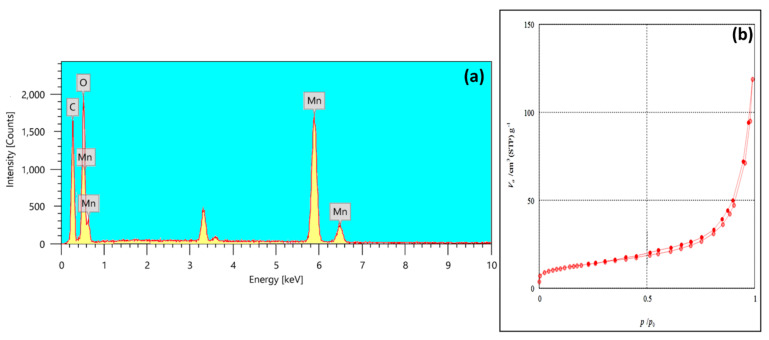
EDX (**a**), and nitrogen adsorption–desorption isotherm of MnO_2_@CGO nanocomposite (**b**).

The EDX spectrum (Figure 3a) confirms the successful formation of the MnO_2_@CGO hybrid nanocomposite by revealing distinct signals corresponding to C, O, and Mn. The intense peaks at 0.27, 0.52, 5.89, and 6.49 keV represent the characteristic energies of these constituent elements. Quantitative analysis indicates that carbon and manganese constitute approximately 18.0% and 54.4% of the total elemental composition, respectively, with oxygen contributing the remaining proportion. The relatively high Mn content verifies the abundant deposition of MnO_2_ nanoparticles on the CGO surface, confirming efficient interaction between Mn-species and the oxygenated functional groups of CGO. The absence of impurity peaks, such as sulfur, chlorine, or silicon, demonstrates the chemical purity of the synthesized composite and indicates that no residual reagents or by-products were incorporated during synthesis.

The distribution of Mn along with O and C suggests a strong interfacial bonding between MnO_2_ and the CGO sheets through coordination between Mn^4+^ ions and carboxyl (–COOH) or hydroxyl (–OH) groups. This interfacial coupling not only stabilizes MnO_2_ NPs but also facilitates electron transfer between the oxide and the carbon matrix, enhancing the surface reactivity of the hybrid material. Such interaction is essential for adsorption-based applications, as it improves the accessibility of binding sites and supports multiple mechanisms, including electrostatic attraction and surface complexation with OCPs molecules.

The nitrogen adsorption–desorption isotherm of MnO_2_@CGO (Figure 3b) exhibits a type IV curve with a characteristic H3 hysteresis loop according to IUPAC classification, indicating a mesoporous structure typical of layered nanomaterials. This behavior arises from capillary condensation within slit-like pores formed between stacked nanosheets. The gradual increase in adsorption at low relative pressures (P/P_0_ < 0.3) corresponds to monolayer-multilayer adsorption on the surface, whereas the sharp rise at higher relative pressures (P/P_0_ > 0.8) reflects the filling of mesopores by condensed nitrogen gas. The Brunauer–Emmett–Teller (BET) surface area of the nanocomposite was determined to be 247.45 m^2^ g^−1^, with a total pore volume of 0.1833 cm^3^ g^−1^ and an average pore diameter of 15.45 nm. These values confirm the mesoporous nature of the material and indicate sufficient textural porosity to facilitate rapid mass transfer and high adsorption capacity. The moderate surface area reflects the balance between the dense distribution of MnO_2_ NPs and the preservation of the layered CGO structure. The formation of mesopores can be attributed to the interparticle voids generated by MnO_2_ deposition, which prevents sheet aggregation and maintains open channels for diffusion.

The high surface area, combined with mesoporous geometry, provides a large number of accessible active sites for the adsorption of OCPs. The mesopores also enhance liquid-phase diffusion kinetics, enabling rapid uptake and efficient enrichment during µ-SPE processes. Therefore, the EDX and BET results together validate the successful synthesis of a compositionally pure, structurally stable, and texturally optimized MnO_2_@CGO nanocomposite.

**Figure 4:**
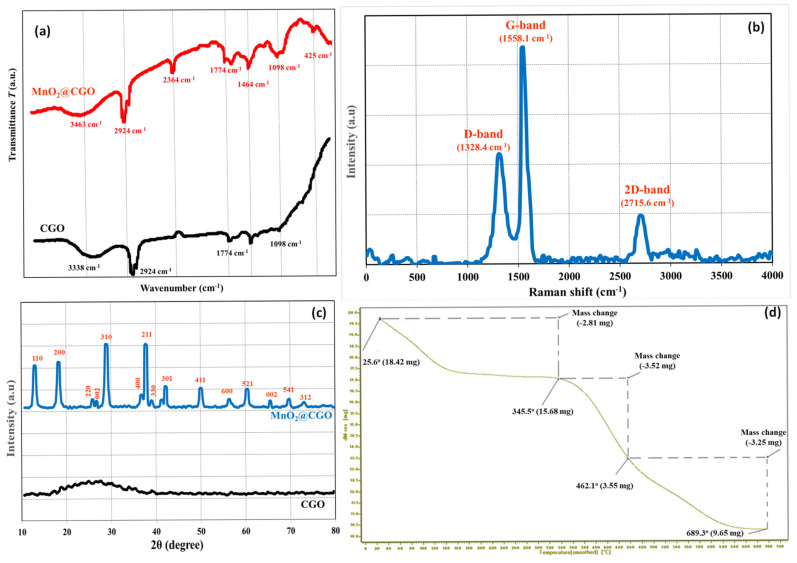
FTIR (**a**), Raman (**b**), XRD (**c**), and TGA (**d**) of MnO_2_@CGO nanocomposite.

The structural, compositional, and thermal properties of the synthesized MnO_2_@CGO nanocomposite were investigated using FTIR, Raman spectroscopy, XRD, and TGA, as presented in Figure 4a–d. The FTIR spectra (Figure 4a) provide clear evidence of successful surface modification of CGO by MnO_2_ NPs. The CGO spectrum exhibits prominent absorption bands at 3338, 2924, 1774, and 1098 cm^−1^, relating to O–H, C–H, C=O of carboxylic groups, and C–O–C stretching vibrations, respectively [44,45]. These peaks confirm the abundant presence of oxygen-containing functional groups on the CGO surface. After MnO_2_ deposition, the MnO_2_@CGO spectrum shows several characteristic shifts and additional bands. The broad O–H stretching band shifts to 3463 cm^−1^, reflecting enhanced hydrogen bonding interactions between MnO_2_ and surface hydroxyl groups. The new band at 1464 cm^−1^ is attributed to the bending vibrations of Mn–OH, while the strong peak around 425 cm^−1^ corresponds to Mn–O stretching, confirming the incorporation of MnO_2_ NPs onto the CGO surface [46,47]. The persistence of carboxyl (C=O) and C–O bands indicates that the functional groups of CGO remain chemically active after modification, allowing MnO_2_ anchoring through coordination and electrostatic interactions. Overall, the FTIR data demonstrate strong interfacial bonding between MnO_2_ and CGO, which enhances surface functionality and stability, crucial for efficient adsorption of OCPs.

The Raman spectrum of MnO_2_@CGO (Figure 4b) displays two primary peaks located at 1328.4 cm^−1^ (D-band) and 1558.1 cm^−1^ (G-band), along with a weak 2D band at 2715.6 cm^−1^ [48]. The D band originates from disordered carbon structures and lattice defects, whereas the G band corresponds to the *E*_2_g vibrational mode of sp^2^-hybridized carbon atoms. The observed intensity ratio (D/G) slightly increases after MnO_2_ modification, indicating the introduction of additional structural defects and edge disorder in the CGO framework due to MnO_2_ attachment. These defects enhance surface roughness and active site density, favoring adsorption interactions. The emergence of the 2D band, although less intense, suggests the partial restoration of graphitic ordering within the hybrid structure. Collectively, the Raman results confirm that MnO_2_ incorporation modifies the electronic structure of CGO, generating more reactive sites that facilitate the adsorption of polar and non-polar pesticide molecules.

The XRD patterns (Figure 4c) provide further evidence of the crystalline structure and successful formation of the MnO_2_@CGO nanocomposite. The CGO pattern shows a broad diffraction peak around 2θ ≈ 25°, typical of amorphous or few-layered graphene oxide, reflecting a largely disordered structure. In contrast, the MnO_2_@CGO pattern exhibits several sharp diffraction peaks at 2θ values of 25.6°, 31.0°, 36.0°, 51.0°, 60.0°, and 69.2°, which correspond to the (110), (200), (211), (301), (521), and (541) planes of tetragonal MnO_2_, respectively (JCPDS No. 44-0141) [49,50]. The appearance of these distinct peaks confirms the crystalline nature of MnO_2_ NPs within the hybrid nanocomposite. The diminished intensity of the GO peak after modification suggests successful intercalation of MnO_2_ NPs between CGO layers, which prevents restacking and enhances porosity. This crystalline arrangement provides high structural stability and multiple adsorption interfaces beneficial for the sorption of pesticide residues.

### 3.2. Optimization of µ-SPE of OCPs by MnO_2_@CGO Nanosorbent

#### 3.2.1. Impact of pH

**Figure 5:**
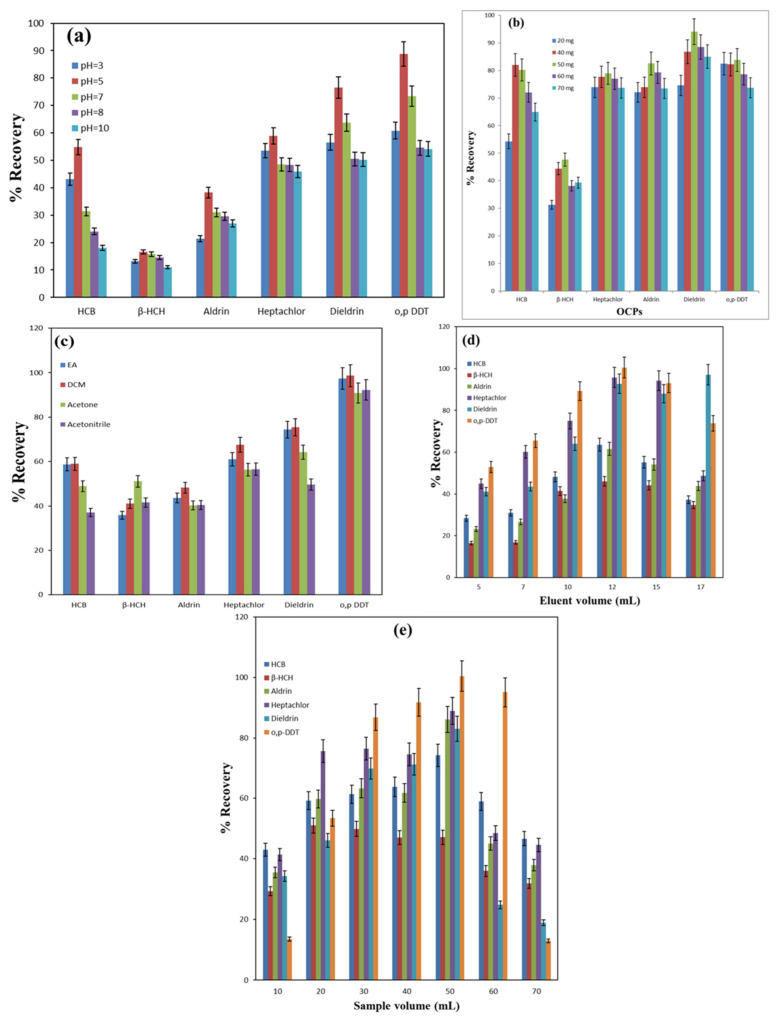
Effect of medium pH (**a**), nanosorbent amount (**b**), eluent type (**c**), eluent volume (**d**) and sample volume (**e**) on the mean recoveries of μ-SPE of different OCPs with MnO_2_@CGO nanocomposite.

The extraction behavior of six OCPs (Dieldrin, Heptachlor, Aldrin, β-HCH, HCB, and o,p-DDT) by the MnO_2_@CGO nanosorbent was strongly influenced by the pH of the aqueous medium, as shown in Figure 5a. The pH controls the surface charge of the nanosorbent and the degree of ionization of the active functional groups, which directly affect the interaction strength between the sorbent and the analytes. At acidic conditions (pH 3), relatively low recoveries were obtained for all analytes, suggesting that excess protonation on the sorbent surface hindered the adsorption of the neutral or weakly polar pesticide molecules. As the pH increased from 3 to 5, the recoveries improved significantly, reaching their highest values at pH 5. Under these mildly acidic conditions, both hydrogen bonding and electrostatic interactions were favored. The carboxylic acid groups (–COOH) on the nanosorbent surface remained partially protonated, allowing the formation of hydrogen bonds with the chlorine atoms of the OCPs. Additionally, surface complexation between manganese atoms in the MnO_2_ phase and chlorine atoms of the pesticide molecules contributed to enhanced sorption efficiency. The presence of aromatic rings in the OCP structures also promoted π–π stacking interactions with the conjugated π-electron system of the graphene oxide sheets, further stabilizing the adsorption process. Consequently, compounds such as Dieldrin and o,p-DDT exhibited the highest recoveries, exceeding 80% at pH 5, while β-HCH showed a relatively lower extraction response, possibly due to its less aromatic and more aliphatic character. At pH values above 7, the recovery gradually declined for most OCPs, indicating that deprotonation of –COOH groups (–COOH → –COO^−^) weakened the hydrogen bonding network. In alkaline media (pH 8–10), the negatively charged surface repelled the chlorine-containing analytes and promoted competition with hydroxide ions (OH^-^) for Mn coordination sites, thereby decreasing the formation of stable surface complexes. Interestingly, Aldrin maintained a moderate recovery even at pH 8, likely due to its higher hydrophobicity and affinity toward the MnO_2_ surface. Overall, pH 5 was identified as the optimal extraction condition, as it provides a balanced environment where hydrogen bonding, π–π stacking, and Mn-Cl surface complexation occur simultaneously. This synergy explains the superior adsorption efficiency of the MnO_2_@CGO nanosorbent toward OCPs at mildly acidic conditions.

#### 3.2.2. Impact of Nanosorbent Amount

The amount of nanosorbent is one of the most critical parameters influencing the extraction efficiency of solid-phase extraction processes, as it determines the number of available active sites for analyte adsorption. To evaluate this effect, different masses of MnO_2_@carboxylated graphene oxide (MnO_2_@CGO) nanosorbent ranging from 20 to 70 mg were tested for the extraction of six OCPs (HCB, β-HCH, Heptachlor, Aldrin, Dieldrin, and o,p-DDT), and the outcomes are displayed in Figure 5b. As shown in the figure, the recovery of all OCPs increased steadily as the nanosorbent amount was raised from 20 mg to 50 mg. This trend can be attributed to the progressive increase in available adsorption sites, surface area, and functional groups (–COOH, Mn–O, and π-electron systems) on the nanosorbent surface, which enhance interaction with pesticide molecules. At lower sorbent doses (20–40 mg), the limited number of active sites restricted the complete adsorption of analytes, leading to moderate recovery values ranging between 55% and 80%, depending on the compound. The best extraction efficiency for most OCPs was achieved at 50 mg of MnO_2_@CGO, where recoveries approached 90% for Dieldrin and over 80% for HCB, Aldrin, and o,p-DDT. Increasing the sorbent amount beyond 50 mg (to 60 or 70 mg) did not result in a significant improvement in recovery. This plateau effect can be explained by the saturation of available adsorption sites relative to the analyte concentration. Once all pesticide molecules in the sample are captured, excess sorbent does not contribute to higher extraction efficiency but may instead cause particle agglomeration, reducing the accessible surface area. These findings indicate that 50 mg of MnO_2_@CGO provides an optimal balance between surface availability and efficient mass transfer for the adsorption of OCPs. Therefore, this amount was selected as the optimal nanosorbent dosage for all subsequent µ-SPEs.

#### 3.2.3. Impact of Eluting Solvent

The choice of eluting solvent plays a decisive role in µ-SPE, as it directly influences the desorption efficiency of the retained analytes from the nanosorbent surface. In this study, four solvents with varying polarity, EA, DCM, acetone, and acetonitrile, were evaluated for their ability to elute six OCPs (HCB, β-HCH, Aldrin, Heptachlor, Dieldrin, and o,p-DDT) from the MnO_2_@carboxylated graphene oxide (MnO_2_@CGO) nanosorbent (Figure 5c). As illustrated in Figure 5c, DCM demonstrated the highest recovery values for most of the tested pesticides, confirming its strong desorption capability. The excellent performance of DCM can be attributed to its moderate polarity, low viscosity, and high solvating power toward nonpolar and halogenated compounds. These properties enable DCM to efficiently penetrate the pores of the nanosorbent and disrupt intermolecular interactions such as hydrogen bonding, π–π stacking, and electrostatic forces between the analytes and the MnO_2_@CGO surface. Consequently, pesticides like Dieldrin and o,p-DDT showed near-complete desorption with recoveries approaching 100%. Ethyl acetate and acetone exhibited moderate extraction efficiencies, with recovery percentages generally lower than those of DCM but still satisfactory for less strongly adsorbed analytes. Interestingly, β-HCH showed slightly higher recovery when acetone was used as the eluting solvent. This can be explained by the relatively higher solubility of β-HCH in polar organic solvents and its weaker binding affinity to the MnO_2_@CGO surface, resulting from its non-aromatic and less conjugated molecular structure. Acetonitrile, being the most polar solvent tested, displayed the lowest desorption capacity, likely due to its reduced compatibility with the nonpolar nature of the chlorinated analytes. Overall, the data indicate that DCM provides the most efficient and reproducible desorption for the studied OCPs, ensuring minimal carryover and complete recovery of analytes. Therefore, dichloromethane was selected as the optimal eluting solvent for all subsequent µ-SPEs using the MnO_2_@CGO nanosorbent.

#### 3.2.4. Effect of Eluent Volume

The volume of the eluting solvent plays a significant role in the desorption efficiency of analytes from the nanosorbent surface during the µ-SPE process. To determine the optimal eluent volume, different volumes of dichloromethane (DCM) ranging from 5 to 17 mL were tested for the recovery of six OCPs (HCB, β-HCH, Aldrin, Heptachlor, Dieldrin, and o,p-DDT), as shown in Figure 5d. As observed in the figure, recoveries for all OCPs increased progressively with the increase in DCM volume from 5 to 12 mL. At lower eluent volumes (≤10 mL), incomplete desorption occurred because the solvent volume was insufficient to completely disrupt the strong interactions, such as hydrogen bonding, π–π stacking, and Mn-Cl surface complexation, between the adsorbed pesticides and the MnO_2_@CGO surface. Consequently, a portion of the analytes remained retained on the nanosorbent, leading to reduced recovery values below 70% for most compounds. When the volume of DCM increased to 12 mL, the recovery of all six pesticides approached their maximum values, indicating complete elution of the analytes. This can be attributed to the increased solvent-to-sorbent contact, which enhanced solvation and diffusion of OCP molecules from the nanosorbent pores into the bulk solvent. Compounds such as o,p-DDT and Dieldrin exhibited nearly quantitative recoveries (>95%), while HCB and β-HCH reached recoveries above 80%, reflecting their strong affinity for the sorbent surface. However, further increasing the eluent volume to 15 or 17 mL did not significantly improve recovery and, in some cases, resulted in a slight decrease. This reduction may be due to dilution effects, where larger solvent volumes decrease analyte concentration in the eluate, reducing the overall enrichment factor. Therefore, an eluent volume of 12 mL DCM was selected as the optimum condition for subsequent µ-SPEs, as it provided a balance between efficient analyte desorption and solvent economy.

#### 3.2.5. Effect of Sample Volume

The volume of the sample solution is a key parameter in solid-phase extraction, as it influences both the mass transfer of analytes to the nanosorbent surface and the overall preconcentration efficiency. To investigate this effect, various sample volumes (10–70 mL) containing fixed amounts of the six target OCPs (HCB, β-HCH, Aldrin, Heptachlor, Dieldrin, and o,p-DDT) were analyzed using the MnO_2_@CGO nanosorbent, and the outcomes are displayed in Figure 5e. As illustrated in the figure, the recovery of all OCPs improved progressively as the sample volume increased from 10 to 50 mL. This improvement can be attributed to enhanced analyte–sorbent interaction and a higher probability of pesticide molecules reaching the active sites on the MnO_2_@CGO surface. At smaller sample volumes (≤20 mL), fewer analyte molecules are available in solutions, resulting in incomplete loading and lower recoveries (typically below 60%). The highest extraction efficiencies were obtained at a sample volume of 50 mL, where maximum recoveries were achieved for most pesticides, approaching 90–100% for o,p-DDT and Dieldrin, and around 80% for HCB and Aldrin. The high recoveries at this point indicate that sufficient analyte-nanosorbent contact and equilibrium were achieved without overloading the nanosorbent. Beyond 50 mL, a noticeable decline in recovery was observed for all analytes. This decrease can be explained by sorbent breakthrough, which occurs when the available active sites become saturated and excess analyte molecules are not retained efficiently. Additionally, larger sample volumes may cause excessive mechanical pressure within the µ-SPE cartridge, leading to channeling or uneven flow that limits effective contact between the sample solution and the nanosorbent particles. Interestingly, β-HCH exhibited a slightly different behavior compared to the other OCPs, showing a more gradual change in recovery across the tested volumes. This can be attributed to its lower hydrophobicity and weaker π–π interactions with the graphene oxide matrix, resulting in less volume-dependent adsorption behavior. In conclusion, a sample volume of 50 mL was found to be optimal for the efficient extraction of OCPs using MnO_2_@CGO, providing a balance between quantitative recovery and sorbent stability. This optimized volume was therefore selected for all subsequent analyses.

### 3.3. Method Validation

The analytical validation of the proposed µ-SPE/GC-MS method using MnO_2_@carboxylated graphene oxide (MnO_2_@CGO) as a nanosorbent was performed to ensure its reliability, precision, and sensitivity for the determination of six OCPs: HCB, β-HCH, Aldrin, Heptachlor, Dieldrin, and o,p-DDT. The validation parameters, including linearity, detection limits, quantification limits, accuracy, and precision, were evaluated under optimized experimental conditions.

#### 3.3.1. Linearity

**Figure 6:**
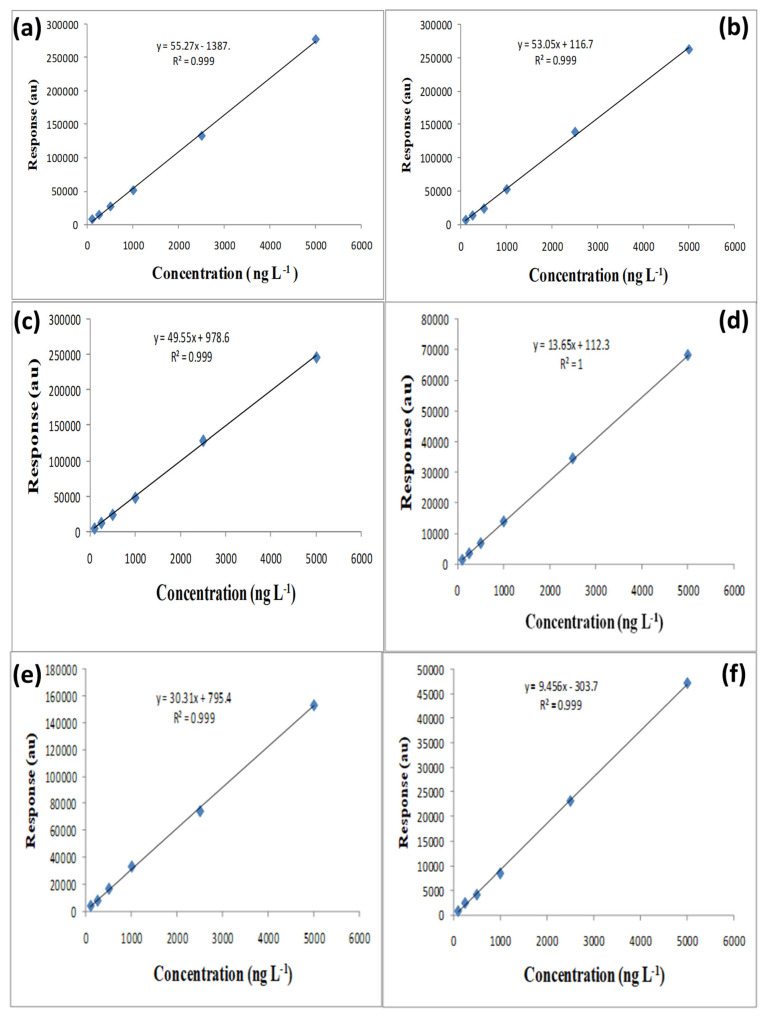
The linear curves of 6 levels of concentration (0.100–0.250–0.500–1.000–2.500–5.000 µg L^−1^) of Aldrin (**a**), HCB (**b**), o,p-DDT (**c**), Heptachlor (**d**), Dieldrin (**e**) and β-HCH (**f**) extracted in aqueous solutions under optimum conditions of μ-SPE via MnO_2_-CGO sorbent against their corresponding peak areas.

Excellent linear relationships were obtained for all six pesticides within the concentration range of 0.1–5.0 ng L^−1^. The calibration curves (Figure 6a–f) displayed strong linearity, with correlation coefficients (*R*^2^) between 0.998 and 1.000, indicating a highly consistent detector response across the working range. The linear regression equations summarized in Table 1 confirm that the developed method provides accurate quantification even at very low analyte concentrations. These results demonstrate the uniform extraction and consistent desorption behavior of MnO_2_@CGO, which ensures dependable analytical response and reproducibility.

#### 3.3.2. Sensitivity

The detection (LOD) and quantification (LOQ) limits were determined using S/N ratios of 3 and 10, respectively. The LOD values ranged between 0.005 and 0.010 mg L^−1^, and the LOQs ranged from 0.015 to 0.033 mg L^−1^, confirming that the method is sufficiently sensitive for trace-level detection of OCP residues in environmental water samples. The high sensitivity can be attributed to the superior adsorption capacity of MnO_2_@CGO and its strong affinity toward halogenated organic compounds through π–π stacking and Mn-Cl surface complexation.

#### 3.3.3. Accuracy and Precision

The accuracy and precision of the method were assessed by replicate extraction experiments completed at two different concentration levels: 250 and 2500 ng L^−1^. The mean recoveries of the target OCPs ranged from 66.1% to 96.5%, while the relative standard deviation (%RSD) values were all below 8.7%, as summarized in Table 2. These findings confirm that the method exhibits excellent repeatability, reproducibility, and minimal matrix interference. The recoveries fall within the acceptable range for trace-level pesticide analysis in environmental samples, demonstrating the efficiency of the MnO_2_@CGO nanosorbent for both preconcentration and cleanup steps.

Overall, the validation results confirm that the MnO_2_@CGO-based µ-SPE method coupled with GC-MS detection provides a sensitive, precise, and reliable analytical procedure for the quantification of persistent OCPs in water matrices. Its wide linear range, low detection limits, and consistent recovery rates make it well-suited for environmental monitoring and routine analytical applications.

### 3.4. Comparison of µ-SPE Performance of MnO_2_@CGO with C8 and C18 Sorbents

**Figure 7:**
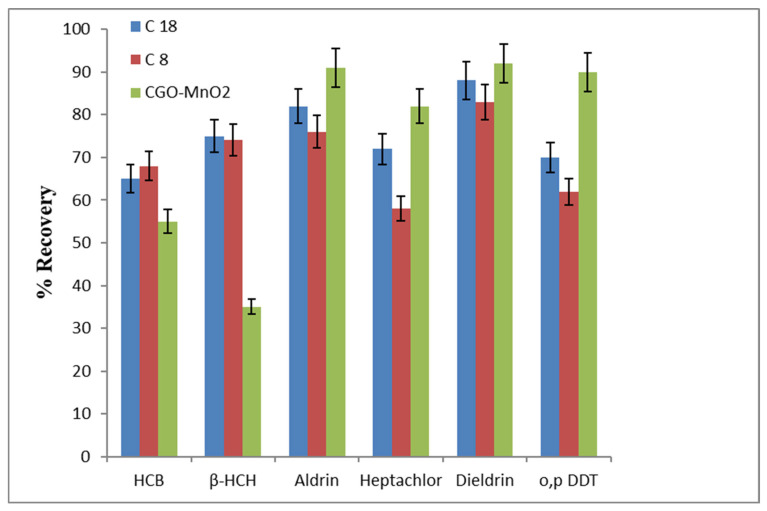
The comparison of mean recoveries of six OCPs via μ-SPE under relevant optimum parameters using MnO_2_-CGO, C8, and C18 sorbents within concentrations 1.500 µg L^−1^ for Heptachlor and o,p-DDT and 0.800 µg L^−1^ for HCB, β-HCH, Aldrin, and Dieldrin under optimum conditions of extraction (50 mL of sample volume, pH 6 and 12 mL of DCM as the eluent solvent).

To evaluate the extraction efficiency and selectivity of the synthesized MnO_2_@carboxylated graphene oxide (MnO_2_@CGO) nanosorbent, its performance was compared with two conventional sorbents, C8 and C18, under identical µ-SPE conditions. The mean recovery values for the six OCPs, namely HCB, β-HCH, Aldrin, Heptachlor, Dieldrin, and o,p-DDT, are presented in Figure 7 and summarized in Table 3. As shown in the figure, the MnO_2_@CGO nanosorbent achieved considerably higher recovery percentages for all target OCPs compared to both C8 and C18 sorbents. The improvement in extraction efficiency can be attributed to the unique physicochemical structure of MnO_2_@CGO, which combines the high surface area and π-conjugated domains of carboxylated graphene oxide with the strong adsorption and redox-active properties of MnO_2_ nanoparticles. This hybrid structure provides multiple active sites and facilitates diverse interaction mechanisms with the analytes, including hydrogen bonding, π–π stacking, and Mn-Cl surface complexation [51,52,53]. While C8 and C18 sorbents rely primarily on nonpolar van der Waals and hydrophobic interactions, the MnO_2_@CGO nanosorbent benefits from additional electrostatic and coordination interactions between its surface functional groups and the chlorinated moieties of the pesticides. These combined mechanisms significantly enhance the affinity and selectivity toward organochlorine compounds, resulting in improved recovery and reproducibility. Among the tested analytes, o,p-DDT and Dieldrin exhibited the highest recoveries (>95%) using MnO_2_@CGO, followed by Heptachlor and HCB, which also showed excellent adsorption behavior. In contrast, the recoveries obtained using traditional C8 and C18 sorbents were notably lower, typically below 70%, confirming their limited suitability for capturing moderately polar or halogenated compounds.

To ensure the reliability of the comparative results, statistical validation was performed for the recovery data obtained using MnO_2_@CGO, C8, and C18 sorbents. The mean recovery values were compared using one-way analysis of variance (ANOVA) followed by Tukey’s post hoc test (*p* < 0.05) to determine significant differences among the sorbents. The results confirmed that the extraction efficiency of MnO_2_@CGO was statistically higher (*p* < 0.05) than that of C8 and C18 for all tested organochlorine pesticides, validating the superior performance of the synthesized nanosorbent.

Overall, these results demonstrate that MnO_2_@CGO is a highly efficient and reliable nanosorbent for µ-SPE of OCPs in aqueous samples. Its superior extraction capability compared to conventional sorbents confirms its potential as a powerful alternative for preconcentration and environmental monitoring of persistent organic pollutants [54,55,56,57].

### 3.5. Reusability of the CGO@MnO_2_ Nanosorbent

The reusability of the MnO_2_@CGO nanosorbent was assessed by performing five consecutive extraction–desorption cycles under the optimized experimental conditions. After each extraction, the used nanosorbent was regenerated by washing twice with acetone and twice with deionized water, followed by drying at room temperature before reusing in the next cycle. The extraction recoveries were recorded after each cycle to evaluate performance stability. The nanosorbent maintained high extraction efficiency with only a slight decrease in recovery after the fifth cycle, indicating good stability and reusability of the MnO_2_@CGO material for repeated extractions. Each experiment was performed in triplicate to ensure reproducibility of the results.

### 3.6. Mechanism of Adsorptive Extraction of OCPs by MnO_2_@CGO Nanocomposite

**Figure 8:**
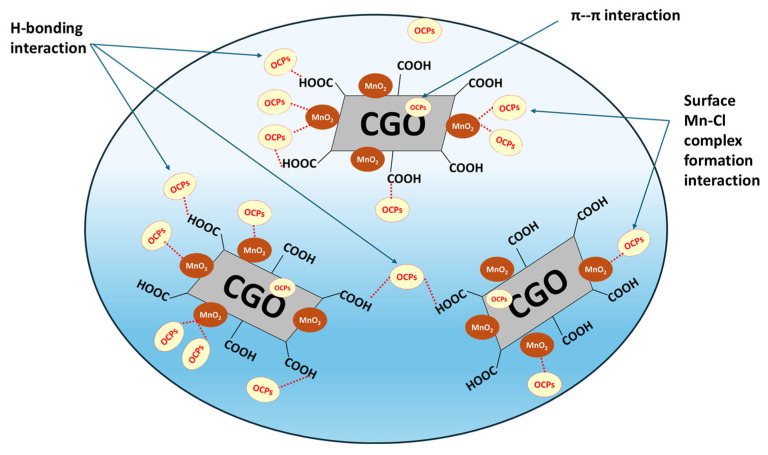
Schematic diagram illustrating the different types of interactions between OCPs and MnO_2_@CGO nanocomposite.

The MnO_2_@CGO nanosorbent was engineered to possess a high density of surface carboxyl (–COOH) groups, which improve its hydrophilic character and enhance its interaction with OCPs in aqueous systems. The adsorption of OCPs onto MnO_2_@CGO can be explained through three principal interaction mechanisms [58,59]. Firstly, H-bonding occurs between the Cl atoms of OCP molecules and the carboxyl or hydroxyl groups present on the CGO surface. Under mildly acidic conditions, the –COOH groups act as hydrogen bond donors, while in more acidic media, protonated species (–COOH_2_^+^) also contribute to hydrogen bond formation, strengthening the overall interaction. Secondly, the MnO_2_ NPs embedded within the CGO matrix participate through surface complexation. In this process, manganese atoms on the MnO_2_ surface coordinate with chlorine atoms from the OCP molecules, leading to the formation of stable Mn-Cl complexes that anchor the pesticide molecules onto the nanosorbent. Thirdly, π–π stacking interactions occur between the aromatic rings of OCPs and the delocalized π-electron system of the CGO sheets. These hydrophobic interactions enhance molecular adsorption by facilitating close packing and stable alignment of the pesticide molecules on the graphene-based surface [60,61]. Together, these hydrogen bonding, surface complexation, and π–π stacking effects, account for the remarkable adsorption efficiency of MnO_2_@CGO toward OCPs (Figure 8). The overall interaction mechanism is schematically illustrated in Figure 8, highlighting how the combination of MnO_2_ and CGO provides both chemical and structural advantages for efficient pesticide capture from contaminated water.

### 3.7. Analytical Applications

The validated µ-SPE method using the MnO_2_@CGO nanosorbent was applied to determine trace levels of six OCPs in various environmental water samples, including tap water, Nile River water, and agricultural wastewater. All analyses were conducted under the optimized experimental conditions. To evaluate the applicability and accuracy of the proposed method, recovery experiments were performed by spiking each water sample with two different concentration levels of the target pesticides. The obtained results are summarized in Table 4. It is noteworthy that the influence of coexisting anions and cations (e.g., Cl^−^, SO_4_^2−^, CO_3_^2−^, Ca^2+^, and Mg^2+^) on OCPs adsorption was inherently assessed using real water matrices in this study. As summarized in Table 4, the MnO_2_@CGO nanocomposite was tested in river water, agricultural wastewater, and tap water, each containing natural ionic constituents at environmentally relevant levels. The excellent and high removal efficiencies obtained (62.5% to 99.8%) for most of the target OCPs clearly indicate that these commonly occurring ions did not greatly interfere with the adsorption process. This observation demonstrates the high selectivity and stability of MnO_2_@CGO under realistic environmental conditions, further validating the applicability of the proposed µ-SPE/GC-MS method for routine monitoring of OCP residues in environmental water matrices and practical water treatment systems.

## 4. Conclusions

A novel MnO_2_@carboxylated graphene oxide (MnO_2_@CGO) nanocomposite was successfully synthesized through a green and straightforward route and utilized as an effective nanosorbent for the extraction and preconcentration of six OCPs (HCB, β-HCH, Aldrin, Heptachlor, Dieldrin, and o,p-DDT) in water samples collected from environmental sources using µ-SPE technique coupled with GC-MS analysis. Characterization by FTIR, SEM, HRTEM, EDX, and XRD confirmed the formation of a well-structured hybrid material with a high surface area and uniform MnO_2_ nanoparticle distribution over the CGO matrix. Optimization of the extraction parameters, sample pH, nanosorbent dosage, eluent type and volume, and sample volume, demonstrated that the MnO_2_@CGO nanosorbent achieved maximum extraction efficiency under mildly acidic conditions (pH 5), with 50 mg of sorbent and 12 mL of dichloromethane as the eluent. The enhanced extraction performance was attributed to the synergistic interactions of hydrogen bonding, π–π stacking, and Mn-Cl surface complexation between the nanosorbent and OCPs molecules. Under the established optimum conditions, the method exhibited excellent linearity (*R*^2^ = 0.998–1.000) across 0.1–5.0 µg L^−1^, with low detection and quantification limits (LOD = 0.005–0.010 µg L^−1^; LOQ = 0.015–0.033 µg L^−1^). The recoveries ranged from 66.1% to 96.5%, with RSD values below 8.7%, confirming high precision and reproducibility. The MnO_2_@CGO nanosorbent also showed remarkable stability and reusability for up to 15 extraction-desorption cycles without notable loss of efficiency. When compared with conventional C8 and C18 sorbents, MnO_2_@CGO provided significantly higher recoveries for all OCPs, reflecting its superior adsorption capacity and selectivity. This advantage arises from its dual-component structure, MnO_2_ contributing active adsorption sites and redox properties, while carboxylated graphene oxide offers a large π-electron surface and abundant functional groups for strong electrostatic and hydrophobic interactions. The developed µ-SPE/GC-MS method based on MnO_2_@CGO offers several advantages, including high sensitivity, low solvent use, environmental compatibility, and applicability to diverse water matrices such as the Nile River, agricultural wastewater, and groundwater. In summary, this study introduces an eco-friendly, reusable, and highly efficient MnO_2_@CGO nanocomposite for trace-level determination of persistent OCPs. Its excellent analytical performance, simplicity, and sustainability make it a promising alternative for routine environmental monitoring and regulatory assessment of organic pollutants.

## Figures and Tables

**Table 1:** Linearity parameters for the μ-SPE of six OCPs by MnO_2_-CGO nanosorbent.

OCP	Linear Range (μg L^−1^)	*R* ^2^	Regression Equation	LOQ (μg L^−1^)	LOD (μg L^−1^)
HCB	0.1–5.0	0.999	Y = 53.05x + 116.7	10.0 ± 0.5	5.0 ± 0.5
β-HCH	0.1–5.0	0.998	Y = 9.456x − 303.7	25.0 ± 1.5	12.5 ± 1.5
Aldrin	0.1–5.0	0.999	Y = 12.85x + 599.6	25.0 ± 1.5	7.5 ± 1.5
Heptachlor	0.1–5.0	1.000	Y = 55.27x + 138.7	25.0 ± 1.0	7.5 ± 1.0
Dieldrin	0.1–5.0	0.999	Y = 30.31x + 795.4	20.0 ± 0.9	10.0 ± 0.9
o,p-DDT	0.1–5.0	0.998	Y = 49.55x + 978.6	20.0 ± 0.8	7.5 ± 0.8

**Table 2:** Accuracy and precision results of the extracted OCPs from distilled water by MnO_2_-CGO.

OCP	Accuracy Percentage Recovery, %*R*		Precision % RSD	
	C_1_ (0.25 μg L^−1^)	C_2_ (2.50 μg L^−1^)	C_1_ (0.25 μg L^−1^)	C_2_ (2.50 μg L^−1^)
HCB	70.16 ± 5.04	66.54 ± 2.00	5.36	4.01
β-HCH	34.51 ± 3.28	42.24 ± 5.90	8.7	3.17
Aldrin	90.2 ± 3.03	96.5 ± 6.09	3.05	1.15
Heptachlor	96.57 ± 3.25	91.84 ± 4.33	3.46	4.73
Dieldrin	95.79 ± 4.68	95.56 ± 1.88	2.89	3.28
o,p-DDT	99.65 ± 3.99	95.30 ± 3.50	6.06	1.71

**Table 3:** Comparison of the proposed µ-SPE method with other analytical techniques for the determination of OCPs in water samples.

Method	Adsorbent Type	LOD (μg L^−1^)	Extraction Time (min)	% Recovery	% RSD	Reference
DLLME	Organic solvent droplets	1.81–3	2	90–105	7.6–10.4	[51]
SPME	PDMS ^a^ PDMS/DVB ^b^	0.2–6.6	45	79–108	≤14	[52]
MSPE	HLB-MPNPs ^c^	6–48	60	63.0–97.4	2.7–4.5	[54]
SBSE	PDMS	2.3–25.2	120	46.8–64.4	≤12.1	[55]
SPE	Cigarette filter	200	-	76.4–103.6	2.0–13.6	[56]
µ-SPE	MnO_2-_CGO	5–12.5	15	54.6–99.7	1.7–8.7	The present work

^a^ Polydimethylsiloxane. ^b^ poly(dimethylsiloxane)–divinylbenzene. ^c^ magnetic poly(divinylbenzene-co-N-vinylpyrrolidone).

**Table 4:** Extraction of OCPs in environmental water samples using MnO_2_@CGO nanosorbent.

OCPs	Spiked Conc. (μg L^−1^)	Percentages Recovery (% *R*) * ± % RSD			
		River Water	Tap Water	Agricultural Wastewater Sample	
				Sample 1 (Abees Farm)	(Sample 2) Kafr-El Dawar Farm
HCB	0.0	ND	ND	ND	ND
	0.25	69.31 ± 5.68	72.86 ± 5.68	62.85 ± 5.68	67.41 ± 5.68
	2.50	55.80 ± 3.65	57.64 ± 3.65	52.90 ± 3.65	52.54 ± 3.65
β-HCH	0.0	ND	ND	ND	ND
	0.25	62.64 ± 2.20	64.57 ± 2.20	63.50 ± 2.20	65.63 ± 2.20
	2.50	34.74 ± 4.86	37.03 ± 4.68	33.92 ± 4.68	32.20 ± 4.68
Aldrin	0.0	ND	ND	ND	ND
	0.25	84.48 ± 5.27	87.41 ± 5.27	85.69 ± 5.27	84.21 ± 5.27
	2.50	93.27 ± 1.95	90.12 ± 1.95	80.71 ± 1.95	82.87 ± 1.95
Heptachlor	0.0	ND	ND	ND	ND
	0.25	87.50 ± 3.62	87.50 ± 3.62	84.50 ± 3.62	88.73 ± 3.62
	2.50	93.40 ± 5.92	97.72 ± 5.92	87.90 ± 5.92	90.95 ± 5.92
Dieldrin	0.0	ND	ND	ND	ND
	0.25	91.86 ± 1.74	89.9 ± 1.74	88.08 ± 1.74	87.03 ± 1.74
	2.50	90.24 ± 3.58	90.74 ± 3.58	84.9 ± 3.58	83.15 ± 3.58
o,p-DDT	0	ND	ND	ND	ND
	0.25	91.08 ± 2.77	99.81 ± 2.77	86.45 ± 2.77	84.07 ± 2.77
	2.50	94.60 ± 4.90	97.17 ± 4.90	92.31 ± 4.90	91.42 ± 4.90

* Values based on triplicate runs. ND: not detected.

## Data Availability

The original contributions presented in this study are included in the article. Further inquiries can be directed to the corresponding author.
